# Vacancy Defects in Ga_2_O_3_: First-Principles Calculations of Electronic Structure

**DOI:** 10.3390/ma14237384

**Published:** 2021-12-02

**Authors:** Abay Usseinov, Zhanymgul Koishybayeva, Alexander Platonenko, Vladimir Pankratov, Yana Suchikova, Abdirash Akilbekov, Maxim Zdorovets, Juris Purans, Anatoli I. Popov

**Affiliations:** 1 Faculty of Physics and Technical Sciences, L.N. Gumilyov Eurasian National University, Nur-Sultan 010008, Kazakhstan; usseinov_ab@enu.kz (A.U.); zhanymgul.k@zerek.kz (Z.K.); Aleksandrs.Platonenko@cfi.lu.lv (A.P.); akilbekov_at@enu.kZ (A.A.); mzdorovets@gmail.com (M.Z.); 2 Institute of Solid State Physics, University of Latvia, 8 Kengaraga Str., LV-1063 Riga, Latvia; Vladimirs.Pankratovs@cfi.lu.lv (V.P.); purans@cfi.lu.lv (J.P.); 3 Department of Physics and Methods of Teaching Physics, Berdyansk State Pedagogical University, 71100 Berdyansk, Ukraine; yanasuchikova@gmail.com; 4 Department of Intelligent Information Technologies, Ural Federal University, 620075 Yekaterinburg, Russia

**Keywords:** DFT, β-Ga_2_O_3_, oxygen vacancy, deep donor, p-type conductivity, point defects

## Abstract

First-principles density functional theory (DFT) is employed to study the electronic structure of oxygen and gallium vacancies in monoclinic bulk β-Ga_2_O_3_ crystals. Hybrid exchange–correlation functional B3LYP within the density functional theory and supercell approach were successfully used to simulate isolated point defects in β-Ga_2_O_3_. Based on the results of our calculations, we predict that an oxygen vacancy in β-Ga_2_O_3_ is a deep donor defect which cannot be an effective source of electrons and, thus, is not responsible for n-type conductivity in β-Ga_2_O_3_. On the other hand, all types of charge states of gallium vacancies are sufficiently deep acceptors with transition levels more than 1.5 eV above the valence band of the crystal. Due to high formation energy of above 10 eV, they cannot be considered as a source of p-type conductivity in β-Ga_2_O_3_.

## 1. Introduction

Gallium oxide (β-Ga_2_O_3_), well known for its unique optical and electrical properties, as a semiconductor with a wide band gap (4.9–5.0 eV), has shown a constantly growing interest as a promising material in different fields of power electronics, optoelectronics, and photonics in recent years [1,2,3]. It can be said, that since the beginning of the century, β-Ga_2_O_3_ has undoubtedly been one of the most intensively studied materials for energy devices. This was initially caused by a large expected electric breakdown field (~8 MV/cm) for its most widespread and technologically promising monoclinic β-modification [1].

Later, as a result of extensive studies, β-Ga_2_O_3_ demonstrates a very wide range of different potential applications in high-power field-effect transistors [1], Schottky barrier diodes [4], solar-blind ultraviolet photodetectors [5], scintillator and phosphor materials [6,7,8], efficient photocatalysts [9], and so on.

A deep understanding of the influence of intrinsic point defects is necessary for the successful application of any material, including a semiconductor. Almost always, point defects directly or indirectly affect and determine the doping and compensation processes, the lifetime of charge carriers, the corresponding migration processes, as well as the efficiency of the different luminescence channels. They also contribute to and even determine the diffusion mechanisms responsible for device degradation.

Interaction and mutual influence of vacancies and dopants only complicates the picture and requires detailed and careful consideration. In the case of β-Ga_2_O_3_, it is assumed that oxygen vacancies, being rather shallow donors with an ionization energy of E_D_ ~0.04 eV, determine its behavior of n-type semiconductors [10]. However, many first-principle calculations devoted to the study of oxygen vacancies in β-Ga_2_O_3_ suggest that they are rather deep than shallow donors with charge transition levels below 1 eV from the bottom of the conduction band [11,12,13,14,15,16]. It is worth to noting that the formation energy of vacancies and, consequently, transition levels of energy in the gap strongly depend on the corresponding band gap evaluation. Proper estimation of the band gap is a major point in a way of correct evaluation of transition levels, as shown by Zacherle et al. [12] and Varley et al. [17]. In our previous hybrid B3LYP study on oxygen vacancies in β-Ga_2_O_3_ [18], it was shown that vacancies create deep donor levels and no have contribution to observable n-type conductivity. Other theoretical calculations have shown that, most likely, shallow donors should be attributed to H or Si impurities in β-Ga_2_O_3_ [17]. On the other hand, several other publications reported that Ni-, Zn-, Cu-, and N-doped β-Ga_2_O_3_ demonstrated p-type semiconductor characteristics [14,19,20,21,22,23].

In this paper, we report first-principles DFT calculations of electronic properties of oxygen and gallium vacancies (V_O_ and V_Ga_) in β-Ga_2_O_3_. The calculated defect transition levels and corresponding defect formation energies obtained in this work are of decisive importance for direct explanation of the experimental results. Furthermore, performed detailed analysis of vibrational spectra will allow constructive identification and detection of defect-induced Raman modes, which may represent the only possibility to characterize such defects at the fundamental absorption edge of β-Ga_2_O_3_.

## 2. Computational Methods

Calculations that were performed in this work used the global hybrid functional B3LYP [24], respectively, implemented in the CRYSTALl17 program [25]. The all-electron Gaussian-type basis sets (BS) for Ga (8s-64111sp-41d) and O (8s-411sp) atoms were taken from Refs. [26,27], respectively. The total energy convergence threshold for the self-consistent field (SCF) procedure was chosen at 10^−7^ and 10^−9^ Hartree for both structural and frequency calculations, respectively. For the geometry optimization, the quasi-Newton scheme was used, as implemented in the CRYSTAL17 code. Gradients are estimated every time the total energy is computed, the second derivative matrix (i.e., Hessian matrix) is accordingly constructed from the gradients and updated by BFGS algorithm. Optimization is considered complete when change in energy between steps is below 10^−7^ a.u. and default gradient and displacement criteria are satisfied. The effective atomic charges, as always, were determined using the Mulliken population analysis [28]. To simulate the point defects in a crystal, the periodic model of an extended unit cell (supercell) was used, and calculations were performed on 80 atoms supercell. The required different charge states of the defect were simulated by adding or removing or electrons to the supercell. To maintain electroneutrality, a compensation background charge was used. The integration of the reciprocal space was performed with a Pack-Monkhorst 4 × 4 × 4 grid [29], resulting in 24 k-points.

The formation energy of a defect *D* with a charge *q* in a system *X* is defined as:

(1) $$E_{f}=E_{tot}\left(D\right)-E_{tot}\left(X\right)+\sum_{i}n_{i}\mu_{i}+q\left(E_{F}-E_{V}\right)+E_{corr}$$

where *E_tot_*(*D*) and *E_tot_*(*X*) are the total energies of the system with and without a defect, *n_i_* represents the number of atoms of the element *i* that are removed from the system when a defect is formed (a negative value for *n_i_* means the addition of atoms), and *μ_i_* is the chemical potential of the element *i* that presents the energy of atoms that are removed (or added) into the system when a corresponding defect is formed. The necessary study was carried out for both oxygen-poor and rich conditions, using molecular O_2_ as a dopant source in the gas phase. The fourth term *q*(*E_F_*−*E_V_*) represents a change in the electron energy due to the exchange of electrons and holes with the carrier reservoirs. *E_F_*−*E_V_* is the Fermi energy relative to the maximum of the valence band of a defect-free system. *E_corr_* are corrections which account for a defect–defect interaction and energy offset when the system is charged. We take the chemical potentials of the O_2_ molecule (oxygen-rich condition) and also the metallic Ga (oxygen-poor condition) as a corresponding limiting phase. The fifth term is a Makov–Payne correction term to compensate the artificial interaction between the periodic images of point charges [30].

For determination of the corresponding charge state transition levels for various defects, we used the approximation described by Lany and Zunger [31], based on the previous study by Scherz and Scheffler [32], stating that the transition level is the Fermi energy, at which the formation energy of a charged defect is equal to that of a neutral defect:

$$\begin{array}{ll} E_{tot}\left(D,q\right)-E_{tot} & \left(X\right)+\underset{i}{\sum }n_{i}\mu_{i}+q\left(\varepsilon \left(q/q^{\prime }\right)-E_{V}\right)\\ & =E_{tot}\left(D,q^{\prime }\right)-E_{tot}\left(X\right)+\underset{i}{\sum }n_{i}\mu_{i}+q^{\prime }\left(\varepsilon \left(q/q^{\prime }\right)-E_{V}\right)+E_{corr} \end{array}$$

thus

(2) $$\varepsilon \left(q/q^{\prime }\right)=\frac{E_{tot}\left(D,q^{\prime }\right)-E_{tot}\left(D,q\right)-E_{corr}}{q-q^{\prime }}+E_{V}$$

As usual, the zero energy reference was chosen at the top of the valence band. We considered cases in which an electronic charge is added to the system, i.e., the state *q’* corresponds to a state with an extra electron. Furthermore, *q*+1*e* corresponds to a transition from a neutral state to a negatively charged state, *ε* (0/−1), or vice versa.

## 3. Results

### 3.1. Atomic and Electronic Structure of Bulk β-Ga_2_O_3_

**Figure 1:**
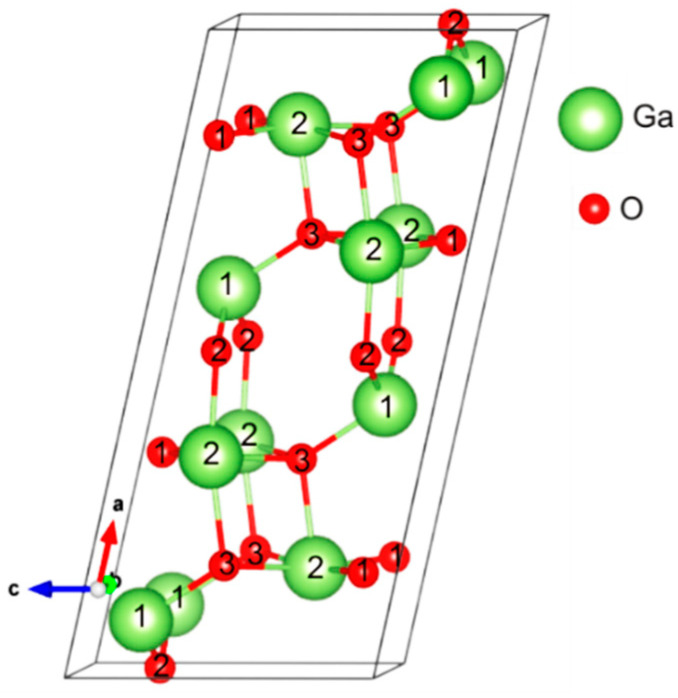
Schematic representation of the crystal structure of monoclinic β-Ga_2_O_3_. Unique positions of Ga and O atoms in the lattice are shown [18].

It is well established that there are five crystalline modifications for Ga_2_O_3_, namely α, β, γ, δ, and ε, among which the β modification is the most stable under ambient conditions [1,2,3]. In particular, β-Ga_2_O_3_ has a monoclinic crystal structure, as commonly denoted as C2/m. The corresponding unit cell of β-Ga_2_O_3_ is shown in Figure 1, (see also [18]), where a⊥c, b⊥c, and the angle between *a* and *c* axes is 104°. The crystal lattice parameters are *a* = 12.19 Å, *b* = 3.05 Å, and *c* = 5.82 Å. It is important that there are two distinct Ga sites, shown as Ga(1) and Ga(2) (Figure 1). The Ga(1) atoms are bonded to four neighboring O atoms in a (roughly) tetrahedral arrangement, while the Ga(2) atoms are in an octahedral environment and are bonded to six neighboring O atoms. Note that not all bonds that extend to atoms in neighboring unit cells are shown in Figure 1. The O atoms have three distinct sites: O(1) and O(2) are bonded to three Ga atoms, while O(3) is combined with four Ga atoms, with the Ga–O bond lengths range from 1.8 to 2.1 Å.

For perfect β-Ga_2_O_3_, the basic bulk properties are calculated (Table 1) using various DFT functionals. As can be seen, the hybrid density functional theory methods give better agreement with experiment in terms of the energy gap *E_g_*, cohesive energy *E_coh_*, and average dielectric constants *ε*, than in the case of the HF method and the standard DFT-GGA functionals, e.g., PBE. The corresponding Mulliken’s analysis performed clearly showed a slight difference in the ionic charge on atoms with different positions in the crystal, which is associated with the anisotropy of the electronic properties [*q*(Ga1) + 1.48*e*, *q*(Ga2) + 1.58*e*, *q*(O1) = −0.994*e*, *q*(O2) = −0.997*e*, *q*(O3) = −0.079*e*], as well as a considerable covalency of the Ga–O bonding (~0.2*e*).

The apparent weakness of indirect transitions and the fact that the corresponding difference in energy between the indirect and direct gap is small actually makes β-Ga_2_O_3_ a direct gap material, which is consistent with the experimentally observed abrupt onset of absorption at 4.9 eV.

The B3LYP-calculated values of the optical dielectric constant *ε*^∞^ give refractive index 1.8 (n = $\sqrt{\varepsilon }$) for β-Ga_2_O_3_ at zero pressure that is in a good agreement with previous theoretical studies and well-known experimental values 1.84 [35], 1.88 [35], and 1.89 [36].

**Figure 2:**
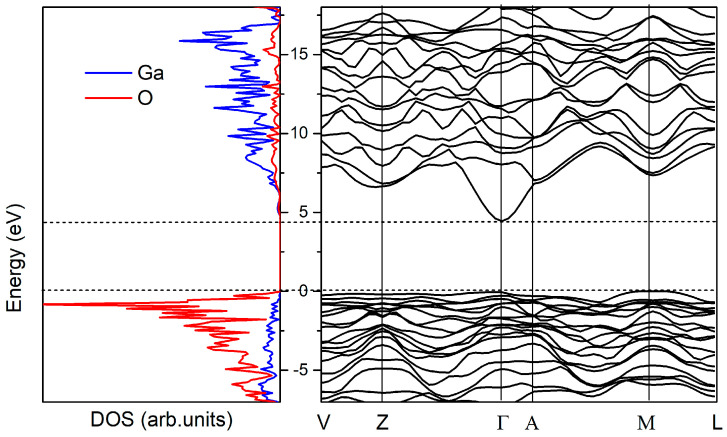
Density of states and band structure of monoclinic β-Ga_2_O_3_, as calculated by means of B3LYP hybrid exchange–correlation functional within DFT in this study.

The calculated density of states and corresponding band structure of β-Ga_2_O_3_ are shown in Figure 2. The top of the valence and the bottom of the conduction band are consisting mainly from anionic O 2p states and cationic Ga 4s states, respectively [10,37]. We find an indirect band gap of 4.89 eV, with the valence band maximum (VBM) located near to the *M-*point, which is slightly smaller than the direct band gap of 4.91 eV at the Γ-point. The corresponding analysis of the dipole matrix elements shows that, although the vertical transitions are of the dipole-allowed type at the Γ point and at the VBM, they are about an order of magnitude weaker at the VBM and quickly decrease to 0 at the *M*-point [38]. The apparent weakness of the indirect transitions and the fact that the corresponding energy difference between indirect and direct gaps is small actually makes β-Ga_2_O_3_ a *direct*-gap material, which is in good agreement with the experimentally observed sharp absorption onset at 4.9 eV [33]. The obtained results of β-Ga_2_O_3_ are in good agreement with previous studies using the pseudopotential plane wave approach [33] and the full-potential linearized augmented plane wave method [12]. Good agreement of the calculated electronic and structural properties with the experimental data gives confidence in the correct prediction of the transition levels of intrinsic defects which often are not achievable from pure DFT methods due to well-known band gap errors.

In order to examine computational setup, we also calculated the elastic properties and frequencies of infra-red (IR) phonon modes of pristine β-Ga_2_O_3_. The elastic constants, bulk modulus, Young modulus and Shear modulus for β-Ga_2_O_3_ are presented in Table 2 with experimental data, while IR-active phonon modes at the Γ-point of β-Ga_2_O_3_ in Table 3. From the group theory analysis we know, that the decomposition of the Reducible Representation at the center of the First Brillouin Zone (k ≈ 0) for β-Ga_2_O_3_ structure is as follows: $\Gamma =10A_{g}+5B_{g}+10A_{u}+5A_{u}$, where three modes are acoustic (*A_u_* + 2*B_u_*), so there are 12 active IR modes and 15 active Raman modes. Both calculations are in good agreement with observed experimental results. The absolute error is higher for modes in the lower part of the spectrum (up to 300 cm^−1^), which mainly correspond to the Ga sublattice bending modes, while Ga–O stretching modes are very well reproduced.

**Figure 3:**
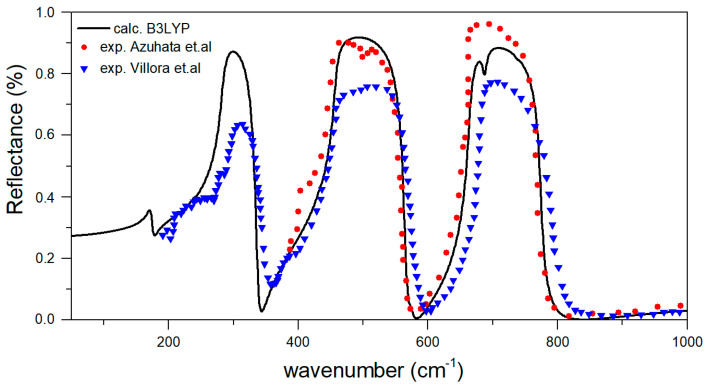
Calculated IR reflectivity for wavevector *q*⊥(100) and specific direction of electric vector E||b. Experimental data are presented for comparison. Experimental data are reproduced from Villora et al. [45] and Azuhata et al. [46] (see text for the details).

The calculated reflection spectrum [44] was computed for direction of electric vector *E* parallel to the *b*-axis (E||b), as shown in Figure 3. As can be seen, the spectrum is comparable with the measured spectra with wavevector *q*⊥(100) and E||b by Villora et al. [45] and even for another direction with *q*⊥(−201) and E||b by Azuhata [46]. This result also confirms the conclusions of Azuhata et al. [46] that pure TO phonons are observed with Au symmetry in the spectrum.

### 3.2. Oxygen Vacancy

**Figure 4:**
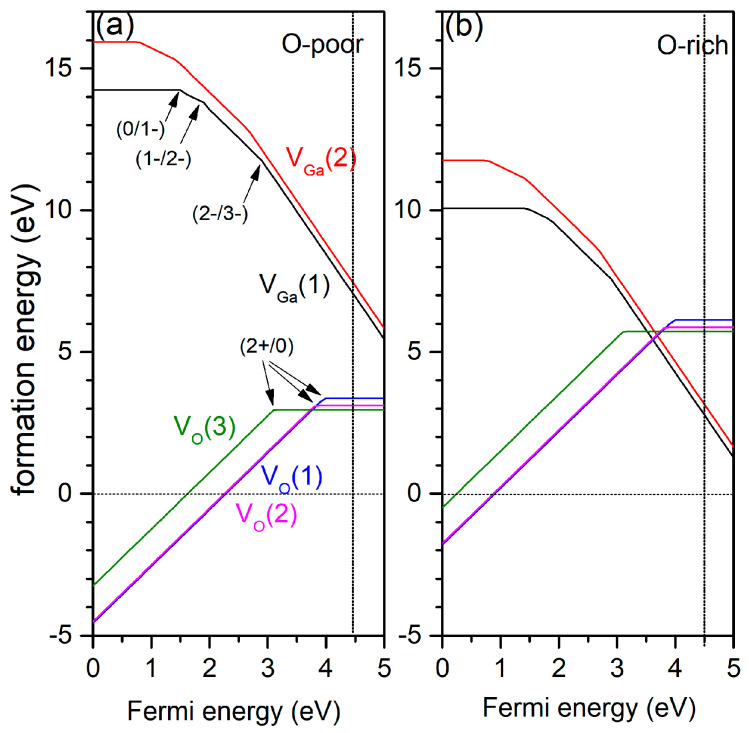
Formation energies of point defects in *β*-Ga_2_O_3_ plotted against the Fermi energy for (**a**) oxygen-poor and (**b**) oxygen-rich conditions. For V_Ga_, two different vacancies are denoted V_Ga_(1) and V_Ga_(2). Analogous notation is used for three oxygen vacancies. The straight vertical line shows the calculated position of the conduction band minimum.

The corresponding formation energies for oxygen vacancies in three different crystallographic positions are shown in Figure 4. Neutral V_O_ has the lowest energy on the O(3) site. In all cases, oxygen vacancy acts as a negative-U defect, where the 1+ charge state is unstable. Obtained transition levels are ε (2+/0) = 4 eV for O(1), 3.8 eV for O(2), and 3.1 eV for O(3) (Table 4). Under oxygen-rich conditions, formation energy increases by ~2.76 eV. As seen, transition levels are located nearly 1 eV under the conduction band minimum (CBM), which is the amount of thermal energy that is much greater than the room temperature to promote an electron from the defect level to the CBM [47]. Therefore, oxygen vacancies are deep donors, i.e., the defects with high ionization barrier and are not the effective source of charge carriers. Meanwhile, as will be shown, further gallium vacancies are deep acceptors with empty levels located high enough from the top of VBM (>0.7 eV) but lower than the donors levels. Thus, oxygen vacancies can easily donate their electrons to gallium vacancies and effectively compensate positively charged defects. As acceptor doping increases, the Fermi level is pushed down toward the VB by decreasing the formation energy of oxygen vacancies as donors. On the other hand, the same process increases the formation energy of acceptors thanks to their charging beginning, thus preventing further reduction in donor formation energy. Thus, it can be assumed that the energy balance can be achieved between these two competing processes when crystal system comes close to equilibrium conditions. Finally, this leads to unchanged concentration of native donors and acceptors.

**Figure 5:**
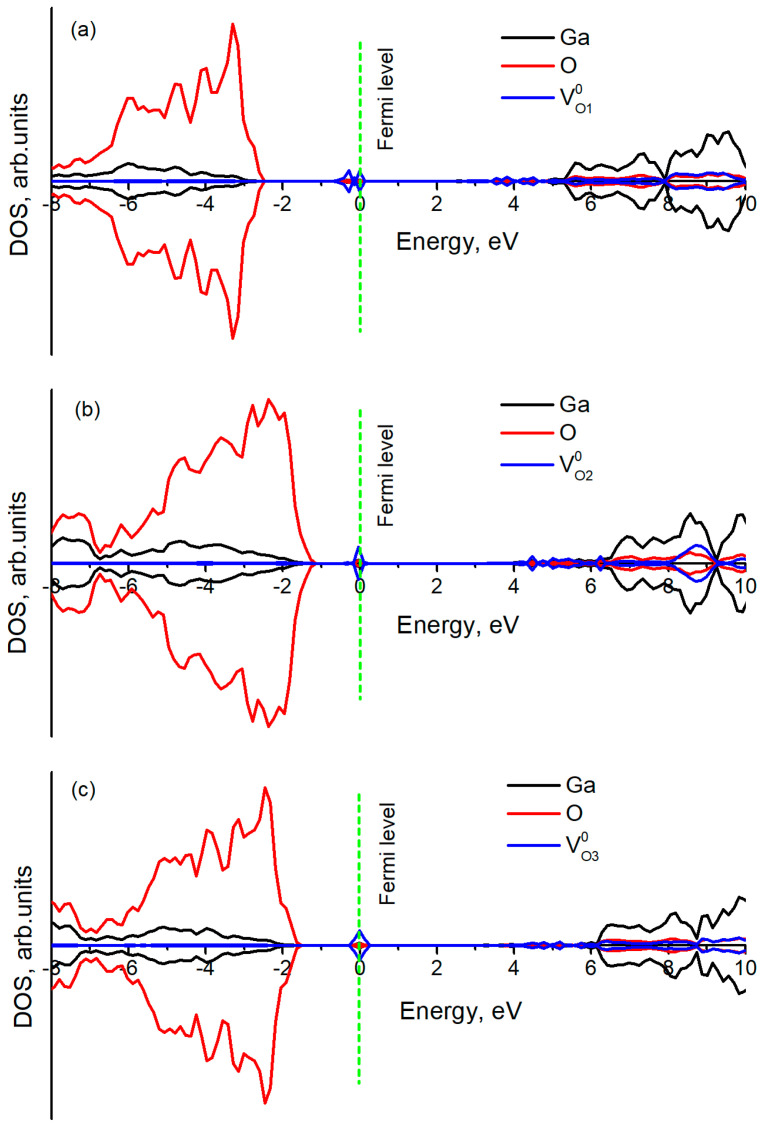
Total density of states for three types of V_O_ in β-Ga_2_O_3_: (**a**) for V_O1_^0^; (**b**) for V_O2_^0^; (**c**) for V_O3_^0^ (see Figure 1). V_O_ states have been magnified by a factor of 20.

As clearly follows from Figure 5, the creation of an oxygen vacancy leads to the appearance of a corresponding defect level in the band gap. Depending on the arrangement of oxygen vacancies in the lattice, their levels occur at different heights from the top of VBM (or depths from CBM bottom). The difference between DOS defect levels confirms the disparity of the local electronic structure of each oxygen vacancy. Occupied defect states (a neutral oxygen vacancy, two electrons in the defect site) are in the middle of the band gap, thus showing the defect’s high ionization potential and proving this defect as a deep donor. Therefore, the locations of the oxygen vacancy level qualitatively correlate with the calculated charge transition levels, e.g., the levels of V_O1_ lie are higher from the top of VBM than the levels of other vacancies, which indicates a more “shallow donor” nature of the defects (Figure 5).

### 3.3. Gallium Vacancy

Gallium vacancy (V_Ga_) is a common defect in β-Ga_2_O_3_. As it is well known, Ga possesses three valence electrons. Therefore, a missing Ga atom leaves behind three dangling bonds that can accept electrons and, thus, V_Ga_ can act as a triple acceptor and compensate donors, reducing free-electron concentration.

The formation energies of V_Ga_ are shown on Figure 3 at low and high oxygen chemical potentials. Indeed, as it was written above, with an increase in the chemical potential of oxygen, the formation energy of V_Ga_ decreases by ~4 eV. Corresponding calculations clearly show that the Ga(1) vacancy has a lower formation energy than Ga(2). The obtained optical transition levels (in eV) for Ga(1)/Ga(2) vacancies are: ε (0/1−) = 1.49/0.74, ε (1−/2−) = 1.9/1.43, ε(2−/3−) = 2.9/2.7. The difference in the formation energies and transition levels can be related to the anisotropy of the local electronic structure of each defect. Indeed, obtained effective charges of neutral Ga(1) and Ga(2) vacancies shows discrepancy: *q*(V_Ga1_) = +0.83*e* vs *q*(V_Ga2_) = +0.95*e*. The essential difference between transition levels of Ga vacancies is obtained in another calculation with HSE06 hybrid functional by Zacherle [12]. The transition levels lie rather high above the VBM.

**Figure 6:**
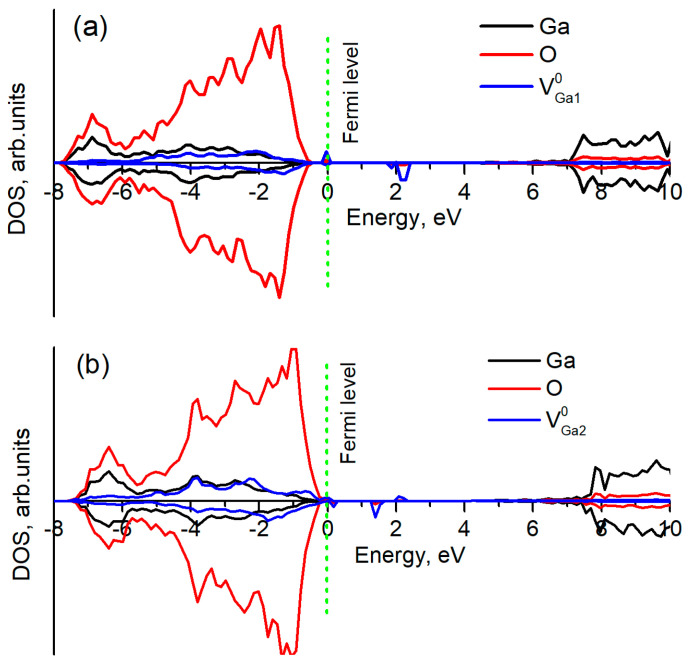
Total density of states for two types of V_Ga_ in β-Ga_2_O_3_: (**a**) for V_Ga1_^0^ and (**b**) for V_Ga2_^0^. Three holes of V_Ga_ are localized on neighboring oxygens.

The calculated density of states for V_Ga_^0^ (Figure 6) also shows that defect states (three holes on oxygen atoms around a vacancy) lie more than 1 eV above the top of valence band, making this defect a deep acceptor; therefore, these vacancies can serve as compensators for donor impurities only. Meanwhile, the formation energy of a neutral vacancy even at O-rich conditions equals more than 10 eV thus, the concentration of such defects under equilibrium conditions is negligible.

For comparison, we summarized transition levels of all vacancies in Table 4 with available literature data. One can see that our results are slightly higher in energy for oxygen vacancies than in the case of the hybrid HSE06 calculations of Zacherle et al. [12] or Varley et al. While, for gallium vacancies, our obtained transition energies are slightly lower. Nevertheless, obtained charge transition levels for all type vacancy defects are comparable among hybrid calculations. Recently, a semi-empiric DFTB study of native defects in β-Ga_2_O_3_ have been reported [48], where a very large supercell of 1120 atoms was used. With a 2-nm dimension, a large supercell essentially lowered the finite-size errors, elastic effects, and the band filling effect, which occur from interactions between charged defects. The results of the DFTB study also agree with our results and confirm other early theoretical investigations.

Meanwhile, the persistent n-type conductivity is often attributed to hydrogen incorporation into crystal. It predicted theoretically that hydrogen can be easily accumulated and stored in the crystal due to a small incorporation barrier of about 0.34 eV and strongly contribute to n-type conductivity because the shallow donor [17]. Interestingly, the same behavior of hydrogen has often been observed in other oxides, e.g., ZnO, SnO_2_, In_2_O_3_ [49]. Thus, it is likely that hydrogen impurity could be a “hidden” source, which drastically transforms the non-conductive β-Ga_2_O_3_ to an n-type conductor. Note that hydrogen in oxides can present itself in different forms and have different charge states [50,51,52,53,54], which, in principle, complicates the overall picture for understanding.

## 4. Summary and Conclusions

In this study, we calculated the energetics and electronic structure of a fully optimized 80-atoms supercell of a monoclinic β-Ga_2_O_3_ crystal using the B3LYP hybrid exchange–correlation functional within the DFT approach. The use of the hybrid B3LYP functional makes it possible to accurately calculate the basic properties of pure β-Ga_2_O_3_. In addition, the calculated elasticity tensors and IR-active vibration modes are in good agreement with those measured experimentally and available from other calculations. The corresponding redistribution of the electron charge in ideal β-Ga_2_O_3_ indicates a noticeable covalence of the Ga–O bounds, which can be confirmed by further detailed X-ray or neutron diffraction analysis.

Due to local anisotropy of each defect, the formation energy of vacancy depends on own local electronic structure and contributes to the difference between transition levels. The formation of the oxygen vacancy in β-Ga_2_O_3_ leads to the presence of deep donor defects. That is why oxygen vacancies can be hardly responsible for n-type conductivity in β-Ga_2_O_3_ irrespective of their quite low formation energy. In this respect, we suggest that the n-type conductivity in gallium oxide is observed due to low donor impurity doping (e.g., by hydrogen) under crystal growth conditions. It is important to note that all types of gallium vacancies with different charge states are predicted to be deep acceptors with corresponding transition levels located more than 0.7 eV above the valence band and with a high formation energy of 10 eV. Our calculations also show that gallium vacancies cannot be the defining reason of p-type conductivity in β-Ga_2_O_3_ crystals.

## Figures and Tables

**Table 1:** Bulk characteristics of pure β-Ga_2_O_3_ as calculated by means of the DFT-LCAO method in this study. The lattice parameters *a*, *b*, *c* as well as band gap *Eg* values were calculated earlier and adopted from [18].

	HF	PBE	B3LYP	Exp
*a*, Å	12.19	12.34	12.34	12.12 ÷ 12.34 [2]
*b*, Å	3.05	3.11	3.09	3.03 ÷ 3.04 [2]
*c*, Å	5.82	5.90	5.87	5.80 ÷ 5.87 [2]
*E_g_*, eV	13.8	2.36	4.49	4.9 [33]
*E_coh_*, eV	−4.33	−7.08	−8.36	−11.3 [34]
*ε*^∞^(average)	2.38	3.67	3.14	3.57, 3.38, 3.53 [34]
*ε*^0^(average)	8.06	10.61	9.6	10.2

**Table 2:** Calculated elastic coefficients *c*_ij_, bulk modulus *B_H_*, Yong modulus *E_H_* and shear modulus *G_H_* (all in GPa) for β-Ga_2_O_3_ together with experimental values. The subscript H according to Voigt–Reuss–Hill notation.

	***C*_11_**	***C*_12_**	***C*_13_**	***C*_15_**	***C*_22_**	***C*_23_**	***C*_25_**	***C*_33_**
This work (B3LYP)	235.3	123.9	138	−12.8	357.2	75.8	6.9	356.5
PBESOL [39]	208	118	146	0	335	83	0	318
Exp [40]	238	130	152	−4	359	78	2	346
Exp [41]	243	128	160	−1.6	344	71	0.4	347
	***C*_35_**	***C*_44_**	***C*_46_**	***C*_55_**	***C*_66_**	***B_H_***	***E_H_***	***G_H_***
This work (B3LYP)	12	54.7	15.2	80.9	101.4	179	213.5	82
PBESOL [39]	19	50	9	77	96	171	192	73
Exp [40]	19	49	6	91	107	184	213	82
Exp [41]	1	48	5.6	89	104	183	210	80

**Table 3:** Calculated infra-red active phonon modes (in cm^−1^) at the Γ-point of β-Ga_2_O_3_ crystal along with known experimental data.

Mode	This Work	[42]		[43]
Symmetry	Calc	Calc	Exp	Exp
Au(1)	173	155	154	-
Bu(1)	241	202	213	-
Bu(2)	280	260	262	272
Bu(3)	283	289	279	287
Au(2)	287	327	296	301
Bu(4)	362	365	356	357
Bu(5)	434	446	432	448
Au(3)	457	475	448	486
Bu(6)	573	589	572	573
Au(4)	664	678	663	671
Bu(7)	687	705	692	716
Bu(8)	737	753	743	774

**Table 4:** Transition levels, *ε(q/q’)* (in eV), of vacancy defects in β-Ga_2_O_3_.

Defect	Transition	This Work (B3LYP)	Zacherle et al. (HSE06) [12]	Varley et al. (HSE06) [13,17]
V_O1_	2+/0	4	3	3.31
V_O2_	2+/0	3.8	3.2	2.7
V_O3_	2+/0	3.1	2.4	3.57
V_Ga1_	0/1−	1.49	2	1.65
	1−/2−	1.9	2.3	2.9
	2−/3−	2.9	2.8	3.3
V_Ga2_	0/1−	0.74	1.3	2
	1−/2−	1.43	1.8	2.5
	2−/3−	2.7	2.45	3

## Data Availability

The data presented in this study are available on request from the corresponding author. The data are not publicly available due to the ongoing research.
